# Case report: Acute oxalate nephropathy due to traditional medicinal herbs

**DOI:** 10.3389/fmed.2022.1063681

**Published:** 2022-12-01

**Authors:** Lirui Wang, Zhuxian Zhu, Jiangtao Li

**Affiliations:** Department of Nephrology, Tongji Hospital, Tongji University School of Medicine, Shanghai, China

**Keywords:** acute kidney injury, acute oxalate nephropathy, medicinal herbs, hyperoxaluria, renal biopsy

## Abstract

Acute oxalate nephropathy (AON), defined as the association between acute kidney injury (AKI) and the deposition of oxalate crystals in the renal parenchyma, is a rare complication of hyperoxaluria. We report a rare case of AON in an adult due to medicinal herbs intake leading to crystal-induced AKI. We recommend that a thorough medication history including the use of medicinal herbs, should be obtained for all patients with a rapid loss of kidney function, especially in the absence of known risk factors for AKI. The use of medicinal herbs with unknown oxalate contents would increase the risk of AON and should be avoided.

## Introduction

Oxalate is originated endogenously from the metabolism of amino acids or exogenously from the intake of oxalate-rich foods (1). The kidney is the sole organ responsible for oxalate excretion (2). Excessive oxalate in the kidney causes the formation of insoluble calcium oxalate crystals (3). This, in turn, leads to a spectrum of kidney disorders, including nephrolithiasis, nephrocalcinosis, and acute oxalate nephropathy (AON) (4). AON can be observed with primary hyperoxaluria (PH) (5) and secondary hyperoxaluria (SH) (6), with the first being inborn errors of metabolism and the second a result of enteric hyperoxaluria or excessive oxalate intake.

Here, we describe a case of acute kidney injury (AKI) after excessive ingestion of Chinese medicinal herbs with previously normal renal function, with tubular deposition of oxalate crystals evident on renal biopsy.

## Case

A 25-year-old male patient presented to the emergency room with recurrent episodes of lumbar pain, nausea, and vomiting in the preceding days. The patient was healthy and had no previous medical problems. A family history of urolithiasis was not noted. Laboratory workup revealed non-oliguric AKI with a serum creatinine of 237 μmol/L, elevated from a stable baseline of 84 μmol/L. Post-renal causes were excluded because the renal ultrasound did not show any signs of obstructive nephropathy or intra-renal or ureteral concrements. The patient was transferred to the nephrology department for further evaluation.

Except for the bilateral costovertebral angle and lower abdominal tenderness, the rest of the physical examination findings were unremarkable. Renal function was severely deteriorated with serum creatinine levels of 724 μmol/L and urea nitrogen level of 21.75 mmol/L. Electrolytes showed potassium of 4.86 mmol/L and sodium of 136 mmol/L. Laboratory findings demonstrated that parathyroid functions were within the normal range: calcium 2.41 mmol/L, phosphorus 1.43 mmol/L, and intact parathyroid hormone (PTH) 38.45 pg/ml (15–65 pg/ml). The liver function test results were unremarkable. Serological test revealed normal levels of immunoglobulin, complements and antinuclear antibodies. ANCA-antibodies and anti-GBM-antibodies were negative. His 24-h urine protein collection was normal (0.08 g/24 h), but the urine albumin-to-creatinine ratio was slightly elevated at 8.16 mg/mmol. Abdominal computed tomography (CT) were normal.

**FIGURE 1:**
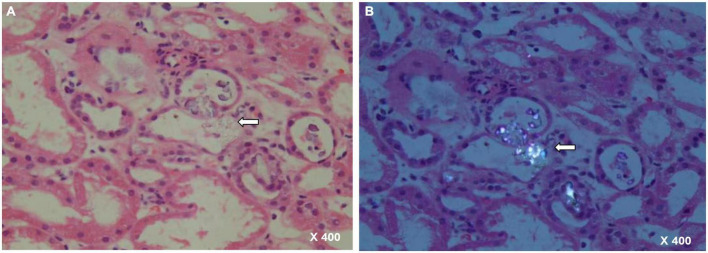
Renal biopsy specimen with arrow showing a couple of calcium oxalate deposits within renal tubules. **(A)** HE staining, 400× magnification. **(B)** Polarized light, 400× magnification.

The calculated fractional excretion of sodium (FeNa) was 1.6% and renal function did not improve upon intravenous volume challenge. As such, pre-renal AKI is unlikely. Since the etiology of AKI remains unknown, a renal biopsy was performed, which showed features of severe acute tubular necrosis without infiltration of inflammatory cells. Abundant tubular calcium oxalate deposits were detected within the tubular lumen (Figure 1). Additional laboratory tests revealed elevated urinary oxalate excretion (1.11 mmol/24 h). Mutations in AGXT, GRHPR, and HOGA1 in PH were not detected (7) (Supplementary Appendix 1).

Upon further questioning, the patient reported an ingestion of approximately 30 g (dry weight) of a mixture of several herbs (*Panax notoginseng, Clematis chinensis Osbeck, Szechwan Lovage Rhizome, borneol, safflower*, and *Eupolyphaga sinensis*) per day in the last 4 weeks before admission for ankle sprain. This traditional remedy is in powder form and mixed with water. He said that the ingestion of such remedies was a customary practice in his hometown. Consumption of vitamin C or ethylene glycol was ruled out.

Elevated urine oxalate levels and renal biopsy led to the diagnosis of oxalate nephropathy, which is the most likely cause of AKI. Given the lack of other explanations, hyperoxaluria was believed to be due to excessive intake of oxalate-rich herbs.

Due to uremic symptoms, emergent hemodialysis *via* a temporary catheter was started before renal biopsy. Totally, three standard hemodialysis were conducted on an Artis hemodialysis machine (Gambro Lundia AB, Lund, Sweden) with a low-flux polysulphon dialyzer F6 (Fresenius Medical Care, Bad Hamburg, Germany). Serum creatinine peaked at 211 μmol/L before plateauing with cessation of dialysis. Once the diagnosis is established, the patient was advised to avoid a high oxalate diet, drink plenty of water (>3 L/1.73 m^2^) and consume calcium acetate (1,334 mg orally with each meal), and potassium citrate (2 g/day, orally). By the day of discharge, renal function had partially recovered, and creatinine levels had decreased to 137 μmol/L. His creatinine level improved and plateaued at 103 μmol/L at 12 months follow-up. Table 1 describes the renal function tests of the patient.

**TABLE 1:** Trend in renal function panel and clinical therapy review.

Timeline line	Day 0 (admission)	Day 1	Day 2	Day 3	Day 5	Day 6	Day 7	Day 10	Day 13 (discharge)	1 months after discharge	3 months after discharge	6 months after discharge	12 months after discharge
Serum creatinine (μmol/L)	724	925			508		211	171	137	101	103	100	103
Blood urea nitrogen (mmol/L)	21.75	21.8			16.54		6.5	6.35	6.8	3.80	4.37	5.72	5.41
eGFR (ml/min/1.73 m^2^)		8.2							60.2	88.3	86.8	89.3	85
**(CKD-EPI equation)**													
Renal biopsy				√									
Oxalate binder therapy					CA #	CA #	CA #	CA #	CA #	CA #			
					PC*	PC*	PC*	PC*	PC*	PC*			
Intermittent hemodialysis			√			√	√						

CA #, calcium acetate (1334 mg orally with each meal); PC*, potassium citrate (2 g/day). Intermittent hemodialysis was conducted using a Artis hemodialysis machine (Gambro Lundia AB, Lund, Sweden) with a low-flux polysulphon dialyzer F6 (Fresenius Medical Care, Bad Hamburg, Germany).

## Discussion

Acute oxalate nephropathy, defined as the association between AKI and the deposition of oxalate crystals in the renal parenchyma, is a rare complication of hyperoxaluria. In the present case, renal biopsy under polarized light revealed massive oxalate crystals in the tubular lumen. This highlights the importance of histopathological confirmations of AON.

Acute oxalate nephropathy can occur due to PH or SH (5, 6). PHs comprise a group of three distinct genetic disorders of glyoxylate metabolism, characterized by endogenous oxalate overproduction (7). Patients with PH typically develop recurrent renal lithiasis and progressive nephrocalcinosis (8). Our patient had no personal history of lithiasis and the genetic testing results were normal. Thus, the clinical picture in our patient was not consistent with a diagnosis of PH.

Secondary hyperoxaluria is more common. The leading cause of SH is enteric hyperoxaluria (9), which is usually a consequence of fat malabsorption (10). In enteric hyperoxaluria, fat malabsorption leads to increased binding of calcium to free fatty acids, resulting in more soluble oxalates in the intestinal lumen, which are subsequently absorbed (11). Enteric hyperoxaluria is often present in patients with inflammatory bowel disease, celiac disease, short bowel syndrome, chronic pancreatitis, or bariatric surgery (12–16). However, our patient showed no clinical symptoms of fat malabsorption.

Increased intake of dietary oxalate or oxalate precursor (such as ethylene glycol or vitamin C) can also contribute to AON (17–20). Prior reports have described patients with oxalate nephropathy due to hyperoxaluria after ingesting oxalate-rich food (17, 18). Dietary sources rich in oxalate include nuts, plums, chocolate, beetroot, strawberries, and spinach (21). Our patient occasionally consumed these foods. In addition, he denied the use of ethylene glycol products or consumption of vitamin C. Therefore, it is unlikely that the dietary oxalate or oxalate precursor was the culprit.

Traditional herbal medicines are naturally occurring, plant or animal-derived substances that are not processed to treat diseases according to local practices. In a study by Huang et al. (22), the total oxalate content of 22 herbs ranged from 165 to 3,204 mg/100 g, which was much higher than that of daily foods such as various flours and nuts. Different medicinal herbs, even those from the same family, contain significantly different amounts of oxalate (22). Oxalate nephropathy has been reported following the consumption of medicinal herbs, such as rhubarb and star fruit (23, 24). In the present case, the absence of evidence for any other cause of AON incriminates the herbal mixture as the culprit. There are no previous case reports in the literature describing oxalate nephropathy in association with any of the involved herbs in our patient. The concentration of oxalate in the same herb may be quite different in different place of origin. Different parts of the same herb, even those from the same place of origin, contain significantly different amounts of oxalate. Due to the lack of detailed information, the concentration of oxalate in each herb was not tested in our study. Thus, it is not known which component of the mixture is responsible for AON. Even so, the findings from our case, at least to some extent, indicate that the use of medicinal herbs with unknown oxalate contents may increase the risk of AON.

We report a rare case of AON in an adult due to medicinal herbs intake leading to crystal-induced AKI. We recommend that a thorough medication history including the use of medicinal herbs, should be obtained for all patients with a rapid loss of kidney function, especially in the absence of known risk factors for AKI. The use of medicinal herbs with unknown oxalate contents would increase the risk of AON and should be avoided.

## Data availability statement

The datasets presented in this study can be found in online repositories. The names of the repository/repositories and accession number(s) can be found in the article/Supplementary material.

## Ethics statement

Ethical review and approval was not required for the study on human participants in accordance with the local legislation and institutional requirements. The patients/participants provided their written informed consent to participate in this study. Written informed consent was obtained from the individual(s) for the publication of any potentially identifiable images or data included in this article.

## Author contributions

ZZ and JL reviewed the medical literature and clinically managed the patients and manuscript. LW reviewed the relevant histopathology and prepared the figure and manuscript. All authors contributed to the conception, drafting, and final approval of the submitted work.
