# Supplementary material for: Case report: Acute oxalate nephropathy due to traditional medicinal herbs
Source: Front Med (Lausanne). 2022 Dec 1;9:1063681. doi: 10.3389/fmed.2022.1063681 (PMC9751358; doi:10.3389/fmed.2022.1063681)
Supplement: Supplementary file 1 [file Data_Sheet_1.PDF]

基因分析报告

样本编号：	19C085084	姓名：	李凌巍	性别：	男
年龄：	24 岁 6 月	样本类型：	外周血	采样日期：	2020-03-12
送检单位：	上海同济大学附属同济医院			病历号：	-
分析项目：	U015：肾结石				
临床诊断：	-				
疾病表型：	-				
重点关注基因：	AGXT|HOGA1|GRHPR				
病史摘要：	腰痛伴血肌酐升高 3 天入院。既往无肾脏病史。				
家族史摘要：	-				
检测方法：	本人-高通量测序				

家系临床信息：

样本标记	样本关系	样本编号	规范化临床表型（CHPO）
李凌巍	本人	19C085084	血清肌酐水平升高,腰痛
李凌巍父亲	父亲	19C149141	-
李凌巍母亲	母亲	19C148960	-

分析结果：

通过对疾病相关基因的测序分析，未发现与疾病表型相关的明确致病性变异

1. 临床表型高度相关，且致病性证据充分的基因变异：未发现

2. 其他变异信息：

基因	染色体位置	转录本外显子	核苷酸氨基酸	纯合/杂合	正常人频率	预测	致病性分析	遗传方式	疾病/表型
CLCNKB	chr1:16375583-16375584[1]	NM_001165945;exon1	c.118delA (p.R40Gfs*4)	het	0.0007	-	Likely pathogenic	1.DR 2.AR	1.双等位基因 Bartter 综合征 4B 型 2.Bartter 综合征 3 型
APRT	chr16:88876892	NM_000485;exon3	c.260G>A (p.R87Q)	het	0.001	D	Uncertain	AR	腺嘌呤磷酸核糖基转移酶缺乏症

注：预测：蛋白功能预测软件 REVEL(rare exome variant ensemble learner)，D：预测为有害；B：预测为良性；-：未知

参考文献： [1] ClinVar:Pathogenic;Bartter syndrome type 3

质控数据统计：

受检者	样本编号	平均测序深度	目标区域覆盖度	
			10X	20X
李凌巍	19C085084	453.37	100.00	99.94

方法学：

测序分析

局限性：

1. 本检测项目只针对已知的与疾病相关的基因，一些尚未明确的基因不在检测范围内。
2. 在数据分析时，为保证数据分析的精确性，目标区域内少部分测序质量过低的变异将被滤掉。
3.此检测方法可以分析到小片段缺失/插入变异，对于更大范围内的缺失/重复，也可以分析，但可信度会降低，需要另一种方法验证，可提供参考结果；NGS 无法检测出所有拷贝数变异，如果怀疑基因存在拷贝数变异，可以进行针对性的 aCGH、SNP array 或 MLPA 分析。
4. 对在捕获区域内的突变，如其所在区域为高 GC 含量区，高度重复序列区，或者在基因组其它位置存在高度同源序列，本方法可能存在一定的假阴性几率
5. 调控区及深度内含子区可能存在的致病性变异无法检测。

注：
1. 如果受检者检测到了自发变异位点，该种情况下并不排除父母生殖腺镶嵌体突变的情况存在，建议遗传咨询。
2. 参考基因组版本为 GRCh37/hg19，核苷酸改变的位置信息是基于当前版本的基因组和转录本，不同基因组版本或不同转录本，这些信息可能会有所不同。
3. hom/het/hemi：hom 表示此突变位点为纯合突变，het 表示此突变位点为杂合突变，hemi 表示此突变位点为半合子突变。
4. MAF：正常人群频率 1000 genomes（千人基因组）、ESP6500（NHLBI Exome Sequencing Project）、EXAC（The Exome Aggregation Consortium）和 EXAC-EAS（EXAC 约 4000 东亚人数据）。
5.蛋白功能预测软件 REVEL(rare exome variant ensemble learner) : Am J Hum Genet. 2016 Oct 6;99(4):877-885.[PMID: 27666373]，D：预测为有害； B：预测为良性； -：未知。
6. 根据美国医学遗传学与基因组学会（ACMG）发布的变异解读指南进行致病性分析：pathogenic/致病性变异；likely pathogenic/疑似致病性变异；uncertain/临床意义未明变异；likely-benign/疑似良性变异；benign/良性变异。本检测只报告根据 ACMG 分级为“致病”、“疑似致病”、“临床意义未明”的变异，不报告“良性”及“疑似良性”等不具有临床意义的变异。有明确家族史的病人，如果有相关家属的基因检测结果会更有助于定性疾病相关的突变。
7. 遗传方式：AR 表示常染色体隐性遗传；AD 表示常染色体显性遗传；XR 表示 X 染色体隐性遗传；XD 表示 X 染色体显性遗传。
8. 基因核苷酸变异的解释是基于目前对该基因的了解，随着研究的深入，这些解释也可能会发生改变。对于某些目前未知临床意义的变异或与其他疾病有关的变异未列在该报告中。

*本报告结果只对送检样品负责。
*以上结论均为实验室分析数据，仅供参考。
*本公司对以上分析结果保留最终解释权，如有疑义，请在收到结果后的 5 个工作日内与我们联系。

实验人员： 李伟阳

分析人员： 王宝霞

北京迈基诺医学检验所 报告专用章

报告签发： 刘志肖

报告日期： 2020-03-27

附录1：分析基因列表

ADCY10	*AGXT*	*ALPL*	*APRT*	*AQP2*	*ATP6V0A4*	*ATP6V1B1*	*ATP7B*
AVPR2	*BSND*	*CASR*	*CBS*	*CDKN1A*	*CDKN1B*	*CDKN2B*	*CDKN2C*
CLCN5	*CLCNKA*	*CLCNKB*	*CLDN16*	*CLDN19*	*CNNM2*	*CTNS*	*CUL3*
CYP24A1	*EGF*	*FAM20A*	*FXYD2*	*GRHPR*	*HGD*	*HOGA1*	*KCNA1*
KCNJ1	*KCNJ10*	*KLHL3*	*LMBRD1*	*MAGED2*	*MEN1*	*MMAA*	*MMAB*
MMACHC	*MMADHC*	*MOCOS*	*MTHFR*	*MTR*	*MTRR*	*MUT*	*NR3C2*
OCRL	*SCNN1A*	*SCNN1B*	*SCNN1G*	*SLC12A1*	*SLC26A3*	*SLC3A1*	*SLC4A1*
SLC7A9	*TRPM6*	*WNK1*	*WNK4*	*XDH*			
